# New therapeutic targets in the treatment of prostate cancer

**DOI:** 10.4103/0970-1591.30270

**Published:** 2007

**Authors:** Vivek Vijjan, Deepak Dubey

**Affiliations:** Department of Urology, Sanjay Gandhi Postgraduate Institute of Medical Sciences, Rae Bareli Road, Lucknow - 226 014, India

**Keywords:** Anti-apoptotic agents, harmone refractory prostate cancer, molecular oncology, prostate cancer

## Abstract

Androgen deprivation therapy has become the mainstay of the treatment of advanced prostate cancer, being used in every clinical setting of the disease, from neoadjuvant to metastatic disease. Despite success in controlling the disease in the majority of men, hormonal manipulations will eventually fail. New agents are being developed for patients with hormone refractory disease. Important advances in molecular oncology have improved our understanding regarding the cellular mechanisms that regulate cell death in the prostate. It is hoped that these new insights will lead to development of more efficacious and easy to tolerate therapies for cancer prostate. This review focuses on the current literature on tumor vaccines, angiogenesis inhibitors, antisense oligonucleotides, differentiation agents, cancer-specific genes, endothelial receptor antagonists, anti-apoptotic agents, agents acting on signaling pathways and androgen and estrogen receptors.

Since Huggins and Hodges first used androgen deprivation for advanced prostate cancer, it has become the mainstay of treatment, being used in every clinical setting of the disease, from neoadjuvant to metastatic disease. Despite success in controlling the disease in the majority of men, hormonal manipulations will eventually fail. The duration of response ranges from nine to 30 months depending on the extent of metastatic disease. Although two recent trials have shown survival benefit for chemotherapy, the optimum time to initiate chemotherapy is still controversial. New agents targeting angiogenesis, apoptosis, signal transduction pathways are being developed for use in combination with chemotherapy.

This review focuses on the emerging targets and the therapies directed at those targets.

## VACCINE THERAPY

Vaccines harness the patient's own immune system to recognize prostate cancer cells and mount a response against them. Major histocompatility complexes (MHC) are present on the on cell surface in the form of endogenously expressed proteins. These tumor os prostate-specific proteins can be the targets of immunotherapy.

PSA is one such prostate-specific target for immunotherapy. It is found in the groove of MHC molecules on the prostate cells. *In vitro* killing of PSA peptide-pulsed cell line as well as PSA-expressing cell line (LNCaP) by human cytotoxic T lymphocytes was demonstrated by Correale *et al*.[1] A Phase 1 trial of patients who failed primary therapy for prostate cancer demonstrated that a vaccinia virus containing the entire human PSA gene could be given with minimal toxicity.[2] Ten patients in this study were given granulocyte/macrophage colony-stimulating factor (GM-CSF) at the vaccine site. The levels of circulating PSA-specific T cells were evaluated in seven patients. Five patients had a doubling of their PSA-specific T cells and four out of these had no PSA progression between six and 11 months.

A second-generation strategy is using priming vaccinations with vaccinia PSA and a vaccinia-encoded T-cell costimulatory molecule (B7.1), which is followed by boosting with fowlpox PSA, all given with GM-CSF and low-dose interleukin-2. A randomized Phase 2 study on patients with HRPC who have no radiographic evidence of disease enrolled 40 patients to either vaccine (n = 20) or nilutamide (n =20) as second-line hormonal therapy.[3] Patients in both arms did better than expected with the median time on study of 10.7 months for the vaccine arm and 10.0 months for the nilutamide arm. There were decreases in PSA levels seen in both arms with one patient in the vaccine arm having a significant sustained decrease in PSA level. There were substantial increases in the numbers of PSA-specific T cells (up to five-fold) as measured by ELISPOT (enzyme-linked immunospot assay) in patients in the vaccine arm.

Chemotherapy has been shown to augment the immune response to a vaccine, perhaps by decreasing the CD4_/CD25_suppressor cells.[4,5] A trial using the above vaccine strategy in combination with weekly docetaxel showed promising immune response both with and without chemotherapy, indicating that an immune response could be achieved, even with chemotherapy and associated steroids.[6]

The GM-CSF gene-transduced prostate cancer cells have been studied to evaluate the clinical effect of combining this potent cytokine with killed tumor cells (GVAX) that have multiple tumor-associated antigens to which the immune system can make a response. A trend toward improved overall survival was observed in a Phase 2 study of this allogeneic prostate cancer vaccine in 34 patients with HRPC (22 vs. 31 months).[7] Dendritic cells (DCs) are considered to be the most potent antigen-presenting cells, in part because of the increased number of costimulatory molecules on their surface. The Pacific Northwest Cancer Foundation incorporated DCs pulsed with prostate-specific membrane antigen (PSMA) peptides in Phase 2 clinical trials.[8–10] The patients received six infusions of DCs pulsed with PSMA-derived peptides, PSM-P1 and PSM-P2, at six-week intervals. Approximately 30% of the patients had a partial or complete response or a >50% reduction in PSA level.

Another active immunization strategy tested a cell product enriched for DCs (APC8015) and pulsed with a PAP–GMCSF construct. A total of 31 patients were enrolled in a Phase 1/2 trial with APC8015 infused four times over 24 weeks.[11] This treatment was tolerated well, immune responses were seen and three patients had a >50% decrease in PSA levels. Provenge, a dendritic cell vaccine, showed activity but failed to produce a significant improvement in time to progression or overall survival, in a Phase 3 trial.[12]

## ANGIOGENESIS INHIBITORS

Neovascular growth into tumors allows not only nutrients and oxygen to flow into the tumor, but provides an avenue of escape for tumor cell metastasis. Microvessel density has been reported to be higher in prostate cancer tissue compared with adjacent hyperplastic or benign tissue. A correlation between increased angiogenesis in primary tumor specimens and the future development of metastatic disease has been observed. Thalidomide, an angiogenesis inhibitor, administered in doses ranging between 200 mg and 1200 mg was studied in 63 patients with metastatic HRPC. A >40% decrease in PSA levels in 27% of patients and improvement in clinical symptoms in all responding patients was observed. The PSA level decreases often coincided with striking reductions in measurable disease on positron emission tomography.[13] Seventy-five patients were enrolled in a trial of docetaxel and thalidomide compared with docetaxel alone in patients with metastatic HRPC.[14] There were decreases in PSA level of >50% in 25 (50%) patients in the combination arm versus nine of 24 (37%) patients in the thalidomide-alone arm. The objective response rate for the two arms was 35% and 27%, respectively. The overall survival was 14.7 months for docetaxel alone versus 28.9 months for the combination (*P*=0.1). The regimen was relatively well tolerated, although prophylactic anticoagulation was recommended to prevent thrombotic complications.

TNP-470, an analog of the *Aspergillus*-derived antibiotic fumagillin, has been shown to inhibit tumor-induced neovascularization. There were no clinical responses seen in a Phase 1 trial of alternate day intravenous (IV) TNP-470.[15] Bevacizumab, a humanized murine monoclonal antibody to vascular endothelial growth factor, has been studied in a Phase 2 study in combination with APC8015 in patients with biochemical failure after definitive therapy. The results revealed that three of nine evaluable patients had a prolongation of the PSA doubling time.[16] In a Phase 2 study the combination of bevacizumab to docetaxel and estramustine in patients with HRPC was studied.[17] Out of 20 patients with sufficient PSA data, a 50% decrease in PSA was observed in 65% of the patients and a partial objective response in nine of 17 patients (53%) with soft tissue disease.

### Antisense oligonucleotides

Antisense oligonucleotides, which are 15 to 25 bases of single-stranded DNA, bind to messenger RNA (mRNA) in a nucleotide sequence-specific manner. This binding inhibits translation of the mRNA, which preferentially undergoes degradation with subsequent inhibition of protein expression and function.

Bcl-2 overexpression is associated with the development of resistance to androgen deprivation and chemotherapy in prostate cancer. A Bcl-2 antisense oligonucleotide (G3139), which binds to the first six codons of Bcl-2, downregulates expression of Bcl-2 and enhances activity of docetaxel and paclitaxel in a murine model.[18] In a clinical trial of G3139 in combination with mitoxantrone in patients with metastatic HRPC, 26 patients were treated with G3139 (0.6 to 5.0 mg/kg per day) as a 14-day continuous IV infusion every 28 days with mitoxantrone (4 to 12 mg/m^2^) given as an IV bolus on Day 8.[19] Two patients had a decrease in PSA >50% and one patient whose mitoxantrone dose was 4 mg/m^2^ had symptomatically improved bone pain. In another Phase 1 trial, 12 patients were treated with G3139 (5 or 7 mg/kg per day) as a five-day continuous IV infusion every 21 days with docetaxel (60 or 75 mg/m^2^) given as an IV bolus on Day 6 of each cycle.[20] PSA decreases were noted in four of eight taxane-naive patients and there were decreases in Bcl-2 protein expression in peripheral blood mononuclear cells by the fifth day of the cycle. A Phase 2 study using higher doses of the two drugs in patients with metastatic HRPC, observed a PSA decrease of >50% in 48% patients, with an objective response in four of 15 (27%) patients with soft-tissue disease. Furthermore, the median decrement of Bcl-2 protein in peripheral blood mononuclear cells at Day 6 of the G3139 infusion was 50%.[21]

Protein kinase C (PKC)-α and *raf*-1 are important elements in the proliferative signal transduction pathways in both normal and malignant cells. PKC-α enhances tumor growth and invasiveness and its expression is upregulated in prostate cancer but not in adjacent normal tissue. ISIS 3521, an antisense oligonucleotide targeting PKC-α and ISIS 5132, an antisense oligonucleotide targeting *raf*-1, were tested in a clinical trial in patients with metastatic HRPC.[22] There were no clinical responses seen in the 31 patients enrolled.

Clusterin is a secreted protein constitutively expressed in most mammalian tissues. Clusterin expression has been shown to increase after androgen ablation, chemotherapy, radiation and other cell stressors. Antisense oligonucleotides to clusterin appear to decrease resistance to these strategies. An antisense oligonucleotide recently introduced into clinical trials is OGX-011 which targets the translation initiation site of the clusterin gene.

## DIFFERENTIATION AGENTS

These agents exploit the cellular machinery to modulate transcription. The cancer cells are reprogrammed to undergo terminal differentiation, conversion to a less malignant phenotype and /or apoptosis. The major classes of differentiation agents under development are retinoids, vitamin D, histone deacetylase (HDAC) inhibitors and peroxisome-proliferator-activated receptor gamma (PPARα) ligands. They exert their effects by acting as ligands for the nuclear receptor superfamily or through interactions with related regulators of transcription. In the absence of ligand, a subset of receptors bind corepressor that suppress transcription by maintaining chromatin in a tightly coiled configuration. After ligand binding, the receptors undergo a conformational change leading to recruitment of coactivator complexes which leads to chromatin unfolding and exposing the target gene promoter.[23]

Retinoids, derived from dietary vitamin A, play a major role in cellular growth and differentiation. Epidemiological studies have demonstrated an inverse association between serum vitamin A levels and the incidence of prostate cancer.[24] Cis-retinoic acid (CRA) has been shown to inhibit cell growth and induce differentiation in LNCaP prostate cancer cell line.[25] All trans retinoic acid (ATRA) was studied in 17 patients with androgen independent prostate cancer (AIPC), in a dose of 50 mg/m^2^ three times a day on days 1 to 14, repeated every 22 days. No clinical responses were seen.[26] In another study on 26 patients with AIPC treated with ATRA 45 mg/m^2^ orally for seven days followed by seven days off, a > 50% decline in PSA was seen in four (15%) patients.[27]

The major reason postulated for little activity of ATRA is due to increased clearance and elimination because of increased activity of cytochrome P-450 enzyme CYP2E1 and N-acetyltransferase in these patients.[28]

Liarozole, a retinoic acid metabolism blocking agent, which increases the effects of endogenous retinoic acid by inhibition of cytochrome P-450 enzyme 4-hydroxylase, has also been studied.[29] A total of 321 patients were randomized to liarozole (n=160) and cyproterone acetate (n=161). Twenty per cent patients in the liarozole group experienced a ≥50% decline in PSA levels as compared to 4% in the cyproterone acetate group. But this difference did not translate into survival benefit, the median survival being 10.3 months in both the groups.

Additive effects of interferon- alpha (IFN-α) and CRA were studied in 16 patients with AIPC. Novel endpoints like, pre and posttherapy biopsies for histology, proliferative index, apoptotic changes and prostate specific membrane antigen (PSMA) immunoreactivity were studied.[30] The combination was well tolerated, but the majority of cases exhibited no gross histological changes and no difference in proliferative or apoptotic indices. However, increased PSMA immunoreactivity was observed, suggesting a potential differentiation effect.

Epidemiological studies have suggested a reduced risk of development of cancer prostate with vitamin D and calcitriol. Preclinical studies demonstrated that calcitriol could induce differentiation, cell cycle arrest and apoptosis in malignant cells. But clinical trials utilizing vitamin D have been limited by calcium-related toxicity. Various strategies employed to overcome calcitriol-related hypercalcemia include the use of alternative doses and schedules, coadministration with corticosteroids or bisphosphonates and the use of vitamin D analogs. In a study of 32 patients with AIPC, calcitriol was given three times weekly in escalating doses of 8-12μg, along with dexamethasone 4mg four times a week.[31] Twenty-one per cent patients achieved a > 50% decline in PSA levels. A combination of calcitriol and zoledronic acid in a dose-escalation protocol failed to reveal a significant decline in PSA or objective radiological responses.[32]

The synergistic effects of a combination of calcitriol and cytotoxic drugs observed in preclinical studies form the basis of recent clinical trials. In a pilot study of 37 patients with castrate prostate cancer, a combination of calcitriol, docetaxel and dexamethasone was studied. A ≥ 50% decline in PSA levels was observed in 30 patients after a median duration of therapy of 10 months.[33]

Histone deacetylation, as a result of the action of HDAC, results in a tightly coiled configuration of chromatin, leading to inhibition of transcription. The HDAC inhibitors cause acetylation of histones resulting in a relaxed configuration allowing exposure of promoter regions and binding of transcriptional machinery. These agents have demonstrated prostate cancer inhibitory effects, both in *in-vitro* and *in-vivo* studies. Suberoylanilide hydroxamic acid (SAHA), phenylacetate and phenylbutyrate are classes of HDAC inhibitors which have entered clinical trials.

PPARγ, a member of nuclear receptor superfamily, is expressed in adipose tissue and plays a key role in the regulation of terminal adipocyte differentiation. PPARγ expression has also been found both in normal and malignant lesions of prostate. However, a high frequency of heterozygous deletions of PPARγ has been demonstrated in prostate cancer. PPARγ ligands like rosiglazone and poiglitazone are currently being studied in various clinical trials.

### Cancer-specific genes

Prostate cancer-specific genes represent a potential target for therapeutic interventions. They may form a basis for extremely precise and effective gene therapeutic approaches directed preferentially to diseased cells.

The most consistently over expressed gene in prostate cancer is prostate cell antigen PCA3 (also known as DD3, abbreviation for differential display code 3). PCA3 is markedly upregulated in cancerous prostate cells, being overexpressed in >95% clinical specimens. In nonmalignant prostate tissue the gene is expressed at an almost negligible level. PCA3 expresses a noncoding messenger RNA (mRNA) and there is no discreet cytoplasmic protein that results from its transcription. The function of this gene is not clearly defined at present.

As even a minute number of PCA3 transcripts can be identified with RT-PCR, quantitative assays have a potential role in the diagnosis and molecular staging of prostate cancer. PCA3 has a potential for use as a screening test for prostate cancer. The only target molecule that can be used is mRNA, because there is no definable peptide product of this gene. The PCA3 mRNA expression is upregulated to an order of 70-fold in prostate cancer as compared to normal benign tissue. In a large clinical study, enrolling 443 men with PSA ≥2.5 ng/ml, screening with a PCA3-based assay had a positive predictive value of 75% and negative predictive value of 84%.[34]

The PCA3 promoter has an important role in gene therapy as it is a very specific marker for prostate cancer. The cancer-specific promoter can be combined with a suicide gene and delivered to the desired cell by an appropriate vector. After entry into the cell, appropriate assembly of the transcription initiation complex occurs, with enhanced elaboration of the therapeutic product resulting in target cell death. Noncancer bystander cells are not destroyed as they lack the cancer-specific products. Preclinical testing of a number of control constructs using PCA3 gene are underway.[35]

### Endothelial receptor antagonists

Endothelin 1 (ET-1) and its receptors ET_A_ and ET_B_ have an important role to play in the biology of prostate cancer, especially the osteoblastic response of bone to metastasis. Acting through ET_A_ receptors, ET-1 appears to be central in cancer-induced osteoblastic lesions.[36] In animal models, the selective ET_A_ receptor antagonist Atrasentan significantly reduced the osteoblastic response occurring from a ET-1-secreting tumor. Thus targeting the osteoblasts may act as a potential strategy for delaying cancer progression.

In Phase 1 clinical trials atrasentan was shown to be safe and well tolerated.[37] Phase 2 trials have shown a delay in time to disease progression in patients receiving atrasentan as compared to placebo. In a recent Phase 3 study examining the role of atrasentan in HRPC with radiological evidence of metastatic disease, there was a significant delay in the time to progression.[38] As the first endothelin receptor antagonist studied in prostate cancer oral atrasentan holds promise for becoming a meaningful agent in the treatment of hormone refractory disease.

### Anti-apoptotic agents

Apoptosis is programmed cell death that results in bundling of cellular contents into apoptotic bodies which are removed by phagocytes. Removal of androgen in normal prostate c4ells results in their death by the process of apoptosis. However, the androgen independent cells have mechanisms to survive the loss of androgen. Two specific proteins are centrally involved in the process of apoptosis, namely caspases and IAPs (inhibitors of apoptotic proteins).

Manipulating the caspases by diethylmaleate (DEM) represents a novel mechanism for increasing their expression and priming the tumor for increased susceptibility to radiation and chemotherapeutic agents.[39] Diethylmaleate acts by depleting the cell of glutathione, thereby inducing apoptosis. This potential area may form the basis of future studies.

IAPs are a group of anti-apoptotic proteins, which protect the cell from various triggers of apoptosis like Fas and TNF-α ligation, Bax-mediated mitochondrial disruption, caspase activation, cytochrome c release, chemotherapeutic agents, radiation and viral infection. IAPs are overexpressed in prostate cancer cell lines and decreasing their expression with antisense oligonucleotides increases apoptotic induction by Fas and TNF.

Thapsigargin, a sesquiterpene lactone derived from the plant Thapsia garganica, is a potent inhibitor of the sarcoplasmic/endoplasmic reticulum calcium ATPase (SERCA) pump. On inhibition there is an elevation in intracellular free calcium resulting in depletion of the pool of calcium in the endoplasmic reticulum resulting in apoptotic death of the cell. To achieve specificity for prostate cells, a prodrug has been synthesized which is specifically cleaved by PSA at the target tissue, thereby releasing the active drug only to the PSA-expressing cells.[40]

## SIGNALING PATHWAYS

A number of signaling pathways govern the emergence of androgen independent prostate cancer.

The epidermal growth factor receptor (EGFR) pathway mitigates proliferative signals induced by EGF and transforming growth factor-α. Increased levels of EGFR have been detected in prostate cancer specimens. The EGFR tyrosine kinase inhibitors, like gefitinib and erlotinib have been developed. Despite encouraging results in preclinical studies, a Phase 2 randomized study had disappointing results in patients with HRPC.[41] None of the 40 patients included had a PSA response or objective response.

Another member of the same family HER-2/neu, has been reported to be present in up to 100% of AIPC samples. But clinical trials with trastuzumab have failed to provide any significant therapeutic benefit.

The PI3K/akt pathway also appears to be dominant in signaling in advanced prostate cancer. This pathway can also be activated by lack of its accompanying inhibitor: the product of PTEN gene. Loss of PTEN has been associated with aggressive histology and advanced stage in prostate cancer. Drugs targeting various levels of this pathway are currently the subject of many trials.

## ANDROGEN AND ESTROGEN RECEPTOR

The androgen receptor (AR) is present on the prostate cell as it undergoes malignant transformation. It can be present even after the malignant epithelium has entered the terminal androgen insensitive stage. The AR may be amplified or mutated in 20-30% of the androgen independent tumors and remains capable of exerting downstream effects after activation by several growth factors. The AR axis represents a potential target for intervention in end-stage disease.

The prostate has two isoforms of the estrogen receptor (ER)-ER-α and ER-α, in the stroma and epithelial cells respectively. ER-α expression is more in high-grade cancers and represents a target for estrogen antagonists to inhibit cell proliferation. Raloxifene, a selective ER modulator, has been shown to inhibit prostate cancer metastasis in rats and can induce apoptosis in human prostate cancer cell lines. In a Phase 2 trial five out of 18 evaluable patients had stable disease and the longest response was 17 cycles.[42]

## CONCLUSION

Important advances in molecular oncology continue to define the basic mechanisms associated with the development, progression, invasiveness and intractability of prostate cancer. These may result in the development of alternative therapies for metastatic hormone refractory disease. While our understanding has improved regarding the cellular mechanisms that regulate cell death in the prostate, a number of questions remain unanswered. Markers other than PSA need to be developed in order to provide insight into the activity of prostate cancer targeted therapies. It is hoped that these new insights will lead to the development of more efficacious and easy to tolerate therapies for cancer prostate.
